# An atypical presentation of a Respiratory Epithelial Adenomatoid Hamartoma, a case report

**DOI:** 10.1016/j.amsu.2019.08.007

**Published:** 2019-09-05

**Authors:** Nisreen Al-Musaileem, Imtiaz M. Qazi, Jassem M. Bastaki, Mahmoud A.K. Ebrahim

**Affiliations:** a Zain Hospital, Ministry of Health, Kuwait City, Kuwait; b Al-Sabah Hospital, Ministry of Health, Kuwait City, Kuwait

**Keywords:** Respiratory epithelial adenomatoid hamartoma, Olfactory nueroblastoma

## Abstract

**Introduction:**

Respiratory Epithelial Adenomatoid Hamartoma (REAH) is a benign disease that can resemble other malignant entities. Thus, it is essential to diagnose it accurately as the treatment approach differs, from radical surgeries in malignant cases, to a simple excision in hamartoma. We present an unusual case of bilateral REAH that was misdiagnosed, and hence it was treated aggressively.

**Case report:**

A 57-year-old male patient presented with anosmia, 2-years history of bilateral nasal obstruction, and was accompanied with a moderate headache. An impression of olfactory neuroblastoma was made after history taking physical examination, and imaging studies. The patient underwent Functional Endoscopic Sinus Surgery (FESS), excisional biopsy of the cribriform plate mass bilaterally, and superior septectomy. Histopathologic examination of the bilateral masses showed sinonasal polyposis with crypting of surface mucosa and pseudoglandular formation. A diagnosis of sinonasal polyps with REAH was established. The patient's nasal obstruction improved, with no recurrence of sinusitis ± polyposis. However, he still complains of anosmia after 2-years follow-up.

**Conclusion:**

Although REAH is a benign disease, it is critical to reach the correct diagnosis, in order to avoid aggressive treatment. Unfortunately, the preoperative investigations were not consistent with REAH, thus it was misdiagnosed and treated aggressively.

## Highlights

• Respiratory Epitheliel Adenomatoid Hamartoma (REAH) is a benign disease that can resemble other malignant diseases.
• The disease is commoner in males and in the third to ninth decades of life.
• The significance of this article is to share our experience with this disease as we did not diagnose the case accurately. Thus the patient was treated aggressively for a malignant case, instead of a simple excision. Hence, one should keep REAH in mind when encountered a similar case to ours.

## 1 Introduction

Hamartomas are benign, non-neoplastic lesions that develop secondary to tissue-development anomalies, and are composed of overgrowth of normal and mature cells and tissues that are normally present in the affected location, but in a disorganized manner [1,2]. Hamartomas were first described by Albrect in 1904 [3]. Although they can arise anywhere in the body, they are more common in the lung, kidney, liver, spleen, and intestine [1].In 1995, Wenig and Heffner described a type of sinonasal tissue anomaly, and designated it as Respiratory Epithelial Adenomatoid Hamartoma (REAH) [4]. The disease is considered a benign entity; however, the histopathological feature can resemble inverted papilloma or well differentiated adenocarcinoma leading to misdiagnosis [5]. Thus, it is essential not to misdiagnose it as a malignant lesion, due to the different treatment approach, from a radical surgery in malignant cases to a simple excision in hamartoma [5]. Herein, we present an unusual case of bilateral REAH, which was diagnosed as olfactory neuroblastoma thus over treated. This case report has been reported in line with the SCARE criteria [6].

## 2 Case presentation

A 57-year-old male patient presented to our Ear, Nose and Throat (ENT) clinic with the chief complaint of anosmia and 2-years history of bilateral nasal obstruction which was reportedly worse on the right side, and was associated with non-specific moderate headache. There was no history of epistaxis, rhinorrhea, recurrent sinusitis, or any neurological complaints. He is a non-smoker. The patient's past medical history was only significant for Type 2 diabetes, controlled with oral medications. Previously he has had no surgeries and no history of any allergies.

ENT examination was unremarkable except for mild non obstructing deviated nasal septum to the right. Nasoendoscopy revealed a mass originating from the roof of the nasal cavity, bilaterally, overlying the olfactory epithelial mucosal surface; the surface of the mass was irregular. Due to nonavailability of the standardized smell testing (University of Pennsylvania Smell Identification Test) at our institution, we were unable to perform such specific testing. Therefore, we used nonirritating substances like grounded coffee, vanilla, and lemon, which stimulate only the olfactory nerve, without causing irritation and stimulation of the trigeminal nerve, as a rudimentary means to test the patient's smell function. The patient was instructed to sniff separately from each nostril while blocking the other nostril, during this time the patient's eyes were kept closed. He couldn't identify the coffee bilaterally and anosmia was confirmed.

**Fig. 1:**
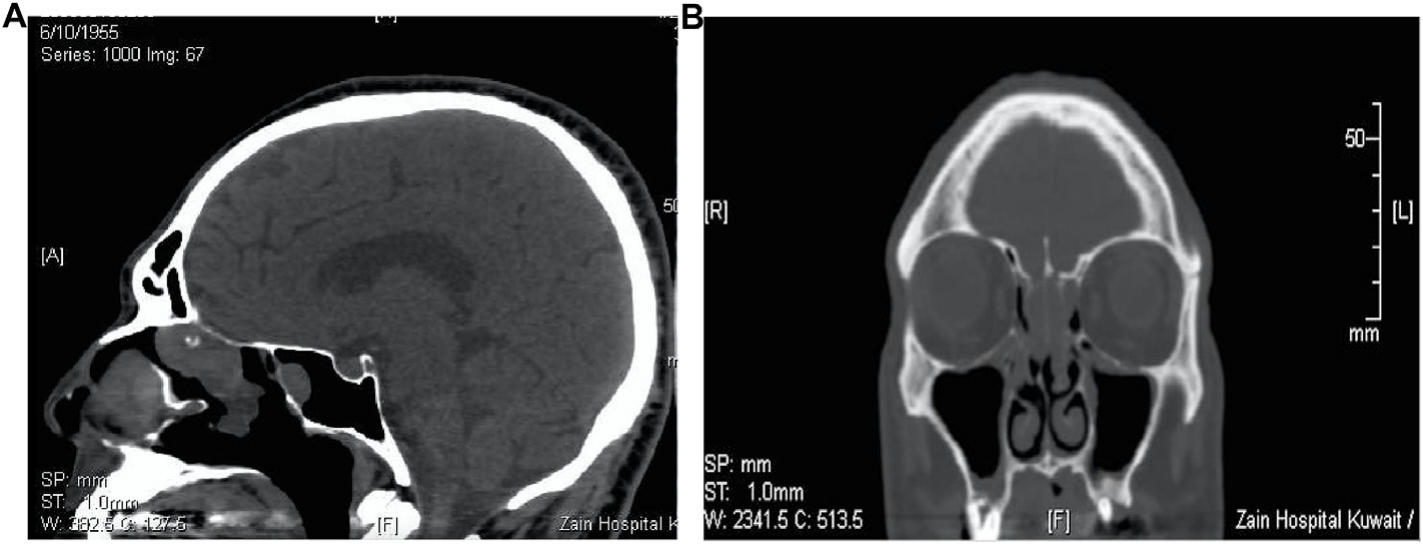
**A & B:** Sagittal (A) and Coronal (B) soft tissue window CT sinus showing bilateral polypoidal soft tissue lesion present at the roof of nasal cavity with intact overlying bony floor of anterior cranial fossa, no intracranial extension on CT basis.

**Fig. 2:**
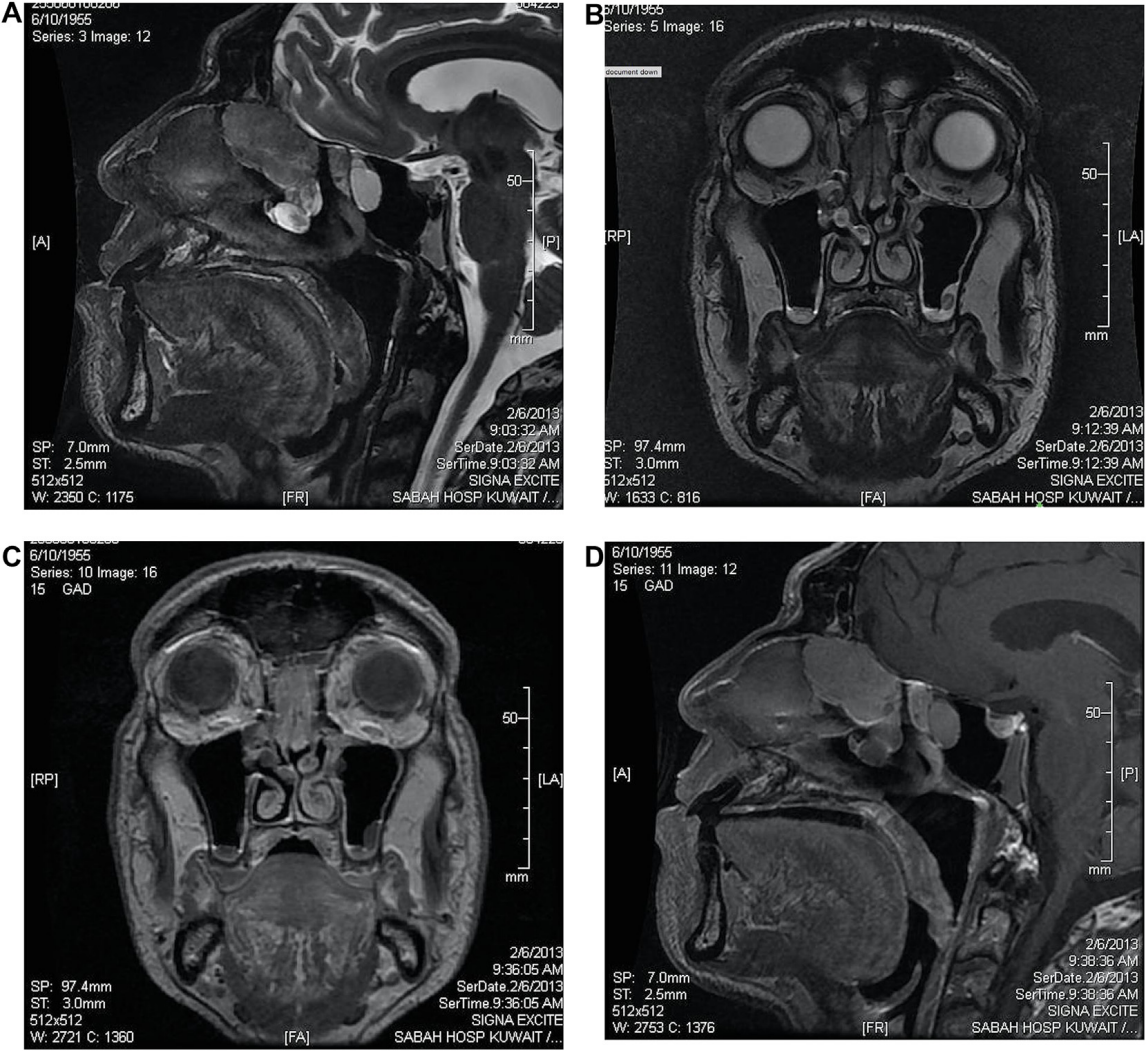
**A, B, C, & D** MRI sinus showing the olfactory recess bilaterally with soft tissue pathology extending to skull base with no intra-cranial extension, showing intermediate signal intensity on T1 post contrast enhancement (A: sagittal, B:coronal) and T2 (C: coronal, D: Sagittal). suggestive of nasal roof mass lesion query neuroblastoma for biopsy.

Routine laboratory investigations were within normal range. Computed Tomography (CT) scan of the nose and paranasal sinuses was carried out which showed bilateral well circumscribed homogenous soft tissue mass at the olfactory recess and polypoid mucosal thickening bilaterally in the maxillary sinuses that was obstructing and dilating the related osteomeatal complexes. The imaging also showed marked polypoid mucosal thickening of ethmoid air cells, sphenoid sinuses and nasal cavities. Also seen was rarefaction of the ethmoidal bony labyrinth and obstruction of the bilateral spheno-ethmoidal recesses as well as an S shaped nasal septum (Fig. 1). The patient was started on topical nasal steroid spray and oral prednisolone; however, the symptoms persisted warranting further investigation of the olfactory mass. The mass was biopsied in the outpatient clinic under topical anesthesia as the mass was well clearly visualized, and was easily accessible with a 45°; the patient was cooperative as well. The histopathology result was inconclusive. Magnetic resonance imaging (MRI) with contrast was ordered (Fig. 2) which revealed a bilateral olfactory recess expansive soft tissue pathology extending to the skull base with no intra-cranial extension, showing intermediate signal intensity on T1 and T2 and post contrast enhancement, which was suggestive of nasal roof mass lesion. These findings were suggestive of olfactory neuroblastoma. The case was discussed in the multidisciplinary team (MDT) meeting, and in view of the inconclusive biopsy report and worrisome radiological findings suggestive of olfactory neuroblastoma, it was unanimously decided to subject the patient to excision biopsy rather than a simple biopsy, under general anesthesia. Subsequently the patient underwent functional endoscopic sinus surgery (FESS) and excisional biopsy of the bilateral cribriform plate mass.

**Fig. 3:**
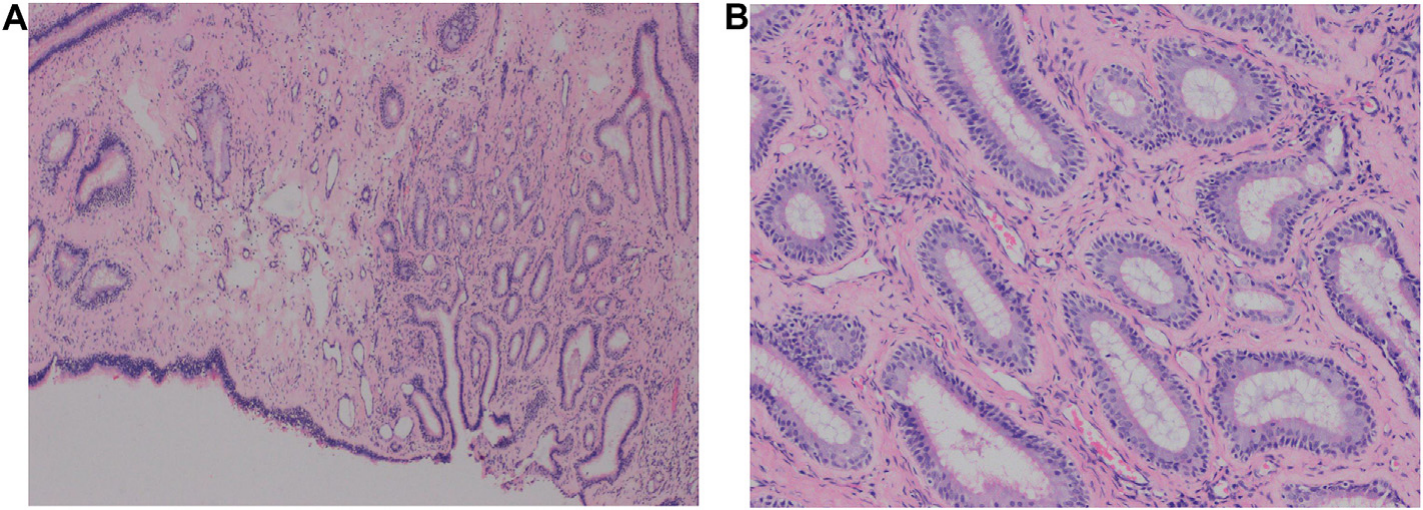
**A & B:** A. Low power (40x) histopathologic photomicrograph showing the surface mucosa ‘crypting’ into the submucosal stroma with gland-like structures masquerading as an infiltrative glandular neoplasm (40x; hematoxylin and eosin H&E). B. Higher power photomicrograph of the lesion illustrating the respiratory epithelium lining those (adenomatoid) structures (100x; hematoxylin and eosin H&E).

The procedure was performed under general anesthesia. Nasal endoscopy revealed a bilateral nasal roof mass at the superior part of the septum and lateral nasal wall, originating from the cribriform plate. The mass was excised from the cribriform plate by making an incision around it. The incision extended 1 cm from the mass to the lateral border of the superior septum and the lateral wall. Although the nasal septum was intact yet a superior septectomy was performed to facilitate en bloc resection of the mass, leaving the cribriform and lateral and medial walls as bare bone. In addition, bilateral FESS, for all four sinuses with removal of the nasal polyps and septoplasty was carried out. Postoperative period was uneventful. Histopathologic examination of the bilateral masses revealed Sinonasal type respiratory epithelium exhibiting submucosal edema, chronic inflammatory cell infiltrate and thickening of the basal layer. These findings are typically seen in cases of sinonasal polyposis; however, prominent crypting of surface mucosa with pseudoglandular formation isn't a typical feature of sinonasal polyps (Fig. 3). A diagnosis of sinonasal polyps with REAH was thus rendered by a subspecialized Head and Neck pathologist.

The patient was followed up in the clinic with serial nasoendoscopy, his nasal obstruction improved gradually; however, he did not regain his sense of smell. The patient is under our follow up for more than two years, his nasal breathing is good, and there is no recurrence of sinusitis ± polyposis. The patient, however, continues to have anosmia. Overall, he is satisfied with his current health status with regards to the nasal disease and our intervention.

## 3 Discussion

Hamartomas are considered non-neoplastic and self-limiting; though, they have no tendency to resolve spontaneously. It is thought that the development of inflammatory polyps leads to the occurrence of REAH [7]. Additionally, an association between nasal polyposis and bilateral REAH of the olfactory cleft has been documented [8]; as is the experience in our patient. We believe that the presence of longstanding bilateral nasal polyposis in our patient led to the development of a bilateral REAH [7]. Complete surgical removal is the standard method of treatment of REAH [9]. Re- emergence of the disease after its resolution is remarkably uncommon [9]. Furthermore, REAH have no malignancy potential [9].

REAHs are uncommon in the head and neck, but the majority of the cases that develop in this region are located in the posterior nasal septum [10]. They typically arise in the third to ninth decades of life, with a male predominance (3:2), similar to the age and gender of our patient, but the location was different in our patient as it originated from the nasal roof [11]. They also tend to occur unilaterally, unlike our case in which it was bilateral [12]. Nasal obstruction, rhinorrhea, epistaxis, recurrent chronic rhinosinusitis, facial pain, and hyposmia/anosmia are some of the symptoms of the disease [9]. Our patient suffered from nasal obstruction, anosmia, and chronic rhinosinusitis.

Two distinctive hypotheses have been proposed regarding the cause of REAH: Congenital and chronic inflammation [13]. According to the chronic inflammatory hypothesis, the development of REAH is a result of chronic and/or severe inflammation of the nasal mucosa in the area of the olfactory cleft [8]. We believe, supported by the long history of chronic sinusitis, that our patient in fact developed REAH secondary to the chronicity of his inflammatory process, and thus fits under the chronic inflammatory hypothesis. Nasoendoscopy findings of REAH are usually of a fleshy to firm polypoid mass. Those findings usually resemble an inflammatory polyp or olfactory neuroblastoma, especially in certain locations, similar to our case [14]. With regards to imaging studies in REAH, there are no specific or diagnostic characteristics [15].

Nasal polyposis, inverted papilloma, and sinonasal adenocarcinoma are the major differential diagnosis contenders of REAH [16]. Inflammatory nasal polyps most often develop from sinus mucosa, whereas REAH usually originate from the nasal septum and olfactory cleft [16]. The histopathological findings of REAH are submucosal proliferation of small to medium-sized round to oval glands lined by ciliated respiratory epithelium, frequently admixed with mucin-secreting cells; while Inflammatory nasal polyps are characterized by stromal edema, epithelial and fibroblast proliferation, and multiple epithelial and subepithelial inflammatory cells infiltrates (eosinophils and lymphocytes), usually devoid of such prominent glandular proliferations. In fact, REAH can sometimes be seen in the background of inflammatory sinonasal polyps [17]. An essential diagnostic method to differentiate between REAH and Nasal polyposis is a CT scan. The CT findings in REAH of the olfactory cleft are classically an opacity of the olfactory cleft that looks enlarged because of the suppression of the middle and superior turbinate to the exterior. In contrast to the findings of nasal polyposis, compression of the middle turbinate can occur against the septum from the exterior [18]. The characteristics of inverted papilloma's are infoldings and invaginations of the mucosal epithelium that are not associated with glandular proliferations, most importantly with prominent neutrophillic microabscesses. Regarding low-grade adenocarcinomas, they appear as a crowded glandular proliferation with pleomorphism, mitoses and different degrees of cellular atypia. In fact, in cases of intestinal type and non-intestinal type adenocarcinomas, both morphology and immunohistochemical studies can help easily differentiate between the two [19].

We present an atypical case of bilateral REAH, in which the clinical, nasoendoscopy, imaging, and the office based biopsy was not consistent with REAH preoperatively. Thus, it was treated aggressively. In hindsight, we believe that the patient should have had a repeat biopsy preferably under general anesthesia, which might have prevented the overaggressive treatment that the patient underwent. Ideally, a simple excision of the mass with a shaver should have been done, instead of excising the whole mass with the surrounding tissue with superior septectomy and leaving bare bone. Hence, one should consider REAH as a differential diagnosis in nasal roof mass with anosmia/hyposmia, chronic sinusitis, rhinorrhea, and nasal obstruction in order to avoid aggressive treatment, such as in our case.

## 4 Conclusion

REAH are benign tumors that can be misdiagnosed due to their less specific clinical and imaging features, which can lead to aggressive treatment, instead of a simple excision. We present a case of bilateral nasal roof REAH that was misdiagnosed due to its atypical presentation and features, consequently it was over treated.

## Ethical approval

Case Reports are exempted from our ethical committee hospital.

## Sources of funding

No source of funding.

## Author contribution

Dr. Nisreen Al-Musaileem: Surgeon of the patient, Came up with the case for the case report, Wrote part of the case report article, analysis of the paper.

Dr. Imtiaz Qazi: Consultant Surgeon of the patient, Edited and proofread the case, Reduced the word count, analysis of the paper.

Dr. Jassem Bastaki: Consultant Pathologist of the case, Substantial editing and proofreading of the article, analysis of the paper.

Dr. Mahmoud Ebrahim: Reviewed the literature, Wrote the introduction + Case + Discussion, Submission process, Corresponding author.

## Conflicts of interest

There is no Conflict of Interest or any financial disclosure.

## Research registry number

1. Name of the registry:
  - Not applicable as it is a case report not a research
2. Unique Identifying number or registration ID:
  - Not applicable as it is a case report not a research
3. Hyperlink to the registration (must be publicly accessible):
  - Not applicable as it is a case report not a research

## Guarantor

Dr. Mahmoud Ebrahim.

Ma7moud_ibrahim@hotmail.com.

Phone: 00965-94445366.

## Provenance and peer review

Not commissioned, externally peer reviewed.
